# Bilothorax as an endoscopic retrograde cholangiopancreatography complication and a review of the literature

**DOI:** 10.1002/rcr2.70035

**Published:** 2024-09-30

**Authors:** Hamza Azam, Mohammed Affan Guliyara, Bapti Roy

**Affiliations:** ^1^ University of Sydney Clinical School of Medicine, Westmead Hospital Westmead New South Wales Australia; ^2^ Department of Respiratory and Sleep Medicine Westmead Hospital Westmead New South Wales Australia; ^3^ Gastroenterology Department Nepean Hospital Penrith New South Wales Australia; ^4^ School of Medical and Health Sciences Edith Cowan University Perth Western Australia Australia

**Keywords:** bilothorax, effusion, ERCP, fluid, pleural

## Abstract

Bilothorax, the accumulation of bile in the pleural space, is an uncommon but serious condition often linked to biliary tract or diaphragmatic injury. This case report describes a 70‐year‐old female with decompensated liver cirrhosis due to primary sclerosing cholangitis, who developed a moderate sized pleural effusion following ERCP and biliary stenting. The patient's pleural effusion persisted for 2 months without respiratory symptoms, indicating a self‐limited low‐volume leak. She eventually underwent thoracentesis for a non‐resolving unilateral effusion, which drained 435 mL of bilious fluid with an elevated pleural fluid bilirubin level, confirming the diagnosis of bilothorax. This case highlights the importance of considering bilothorax as a cause of pleural effusion in patients with biliary tract disease and who undergo high risk procedures including ERCP.

## Graphical abstract

This case report details an unusual presentation of a patient with bilothorax following Endoscopic Retrograde Cholangiopancreatography (ERCP) for biliary stenting. It highlights the importance of considering bilothorax as a cause of pleural effusion in patients with biliary tract disease and who undergo high risk procedures including ERCP.

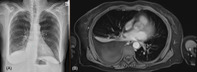

## INTRODUCTION

Bilothorax, also known as cholethorax, is a rare clinical condition where bile accumulates in the pleural space, resulting in a pleural effusion of extra‐vascular origin. Most cases in the literature report bilothorax secondary to biliary tract injury, diaphragmatic injury or secondary to hepatic infections.^1^ The resulting pleural effusion can lead to respiratory distress, necessitating swift diagnosis and intervention to prevent severe morbidity and mortality. Despite its rarity, bilothorax requires heightened clinical awareness for timely recognition and treatment. This case report details an unusual presentation of a patient with bilothorax following Endoscopic Retrograde Cholangiopancreatography (ERCP) for biliary stenting.

## CASE REPORT

A 70‐year‐old female presented with signs of decompensated liver cirrhosis with jaundice, elevated serum bilirubin and ascites. She had a long‐standing history of primary sclerosing cholangitis (PSC) with multifocal stricturing of the biliary tree and Crohn's disease. Her surgical history included an uncomplicated cholecystectomy in 2015. She had no respiratory symptoms and was taking azathioprine and ursodeoxycholic acid for her PSC and Crohn's disease.

Two months prior to the current presentation, she had undergone an ERCP for obstructive jaundice secondary to a common bile duct stricture, treated with a plastic stent insertion. This procedure was subsequently complicated by ascending cholangitis, requiring treatment with intravenous antibiotics. At that time her serum bilirubin was elevated at 140 μmol/L (Reference range: <20 μmol/L). A subsequent ERCP demonstrated stent occlusion, requiring a stent exchange. Following the stent exchange she developed a new cough with a Chest x‐ray (CXR) showing the development of a new small right sided pleural effusion with blunting of the right costophrenic angle.

Examination during the current presentation with decompensated liver cirrhosis, revealed a heart rate of 96 beats per minute. Oxygen saturation was 99% on room air. Her respiratory rate was 14 breaths per minute. She had a low‐grade fever with temperature of 37.5°C. She appeared jaundiced. Abdomen was distended with shifting dullness suggestive of ascites. Chest examination revealed dullness to percussion in the right lung base with reduced breath sounds.

**FIGURE 1:**
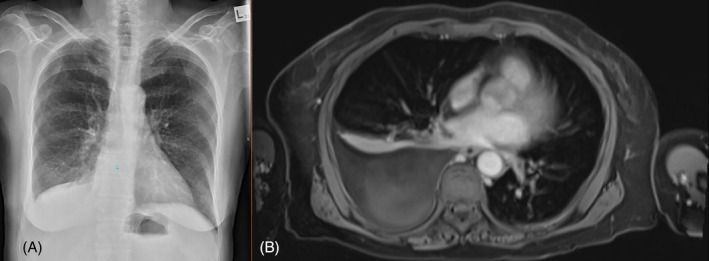
(A) CXR immediately post‐ERCP, revealing a small right pleural effusion with blunting of the right costophrenic angle. The effusion causes obscuration of the diaphragm and a meniscus sign is present, consistent with fluid accumulation in the pleural space. (B) Axial slice from magnetic resonance cholangiopancreatography showing a right pleural effusion.

Blood tests revealed a total serum bilirubin of 248 μmol/L, deranged liver function tests with ALT 119 U/L (Reference range: 10–35 U/L), AST 184 U/L (Reference range: 10–35 U/L), GGT 192 U/L (Reference range: 5–35 U/L), ALP 448 U/L (Reference range: 30–110 U/L). CXR confirmed a small right pleural effusion (Figure 1A), which was unchanged in size from 2 months ago. A Magnetic Resonance Cholangiopancreatography (MRCP) was performed which showed dilated intrahepatic bile ducts with progressive stricturing around the porta hepatis, likely involving the right and left hepatic ducts extending into the common bile duct. A moderate‐sized right pleural effusion was also noted, with no biliopleural fistula or evidence of malignancy (Figure 1B).

**FIGURE 2:**
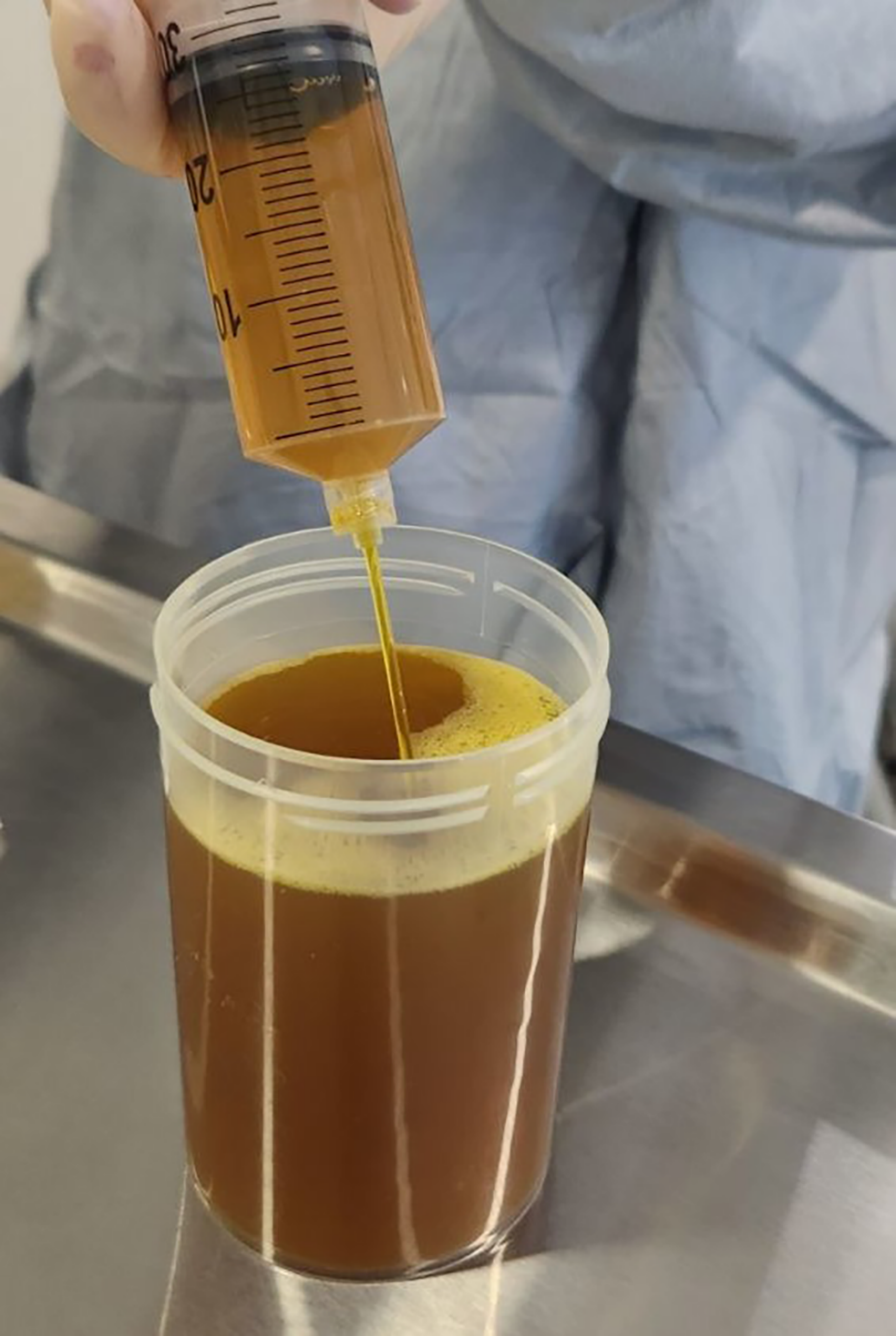
Viscous olive brown pleural effusion sample obtained from diagnostic thoracentesis suggestive of bilothorax.

Given the non‐resolving unilateral pleural effusion, a diagnostic and therapeutic thoracentesis was performed, draining 435 mL of olive brown pleural fluid (Figure 2). The pleural effusion was deemed too small for intercostal catheter insertion and the thoracentesis was sufficient to completely drain the effusion. Pleural fluid analysis confirmed an exudative effusion (Table 1) with a pleural fluid bilirubin level of 170 μmol/L (Reference range: 0 μmol/L). Microscopy and culture were negative and cytology demonstrated reactive mesothelial proliferation with lymphocytosis and no evidence of malignant cells. Results were consistent with a bilothorax, with pleural fluid bilirubin matching that of the serum levels pre‐ERCP, when the effusion first appeared. The patient was commenced on intravenous meropenem while awaiting pleural fluid culture results.

**TABLE 1:** Pleural fluid analysis.

Parameters	Results
pH	7.59
Glucose	7.9 mmol/L
Protein	27 g/L
Bilirubin	170 μmol/L
Albumin	11 g/L
LDH	90 U/L
Amylase	11 U/L
Lipase	19 U/L
Microbiology	Negative
Cytology	Lymphocytosis, no malignant cells

A repeat ERCP was performed and fluoroscopy with radio‐opaque contrast during the procedure did not demonstrate an active biliary leak, suggesting resolution of the initial biliary tree injury. Her Model for End‐Stage Liver Disease (MELD) score was 18. Given progressive disease and no reversible pathology she was transferred to a specialist liver transplant centre for further management. She was deemed eligible for a liver transplant and is currently on the transplant waitlist.

## DISCUSSION

Bilothorax is a rare clinical entity, defined by the presence of bile in the pleural cavity. The passage of bile from the biliary tree into the pleural space can occur through congenital or traumatic defects in the diaphragm (similar mechanism to hepatic hydrothorax), passage between pleuroperitoneal lymphatics, biliopleural fistulas secondary to trauma, infection or iatrogenic aetiology, including operative complications as seen in our case.^2^ Bilothorax was first reported in the literature in 1971 in a male patient following blunt trauma.^3^ Since then, there have only been approximately 100 documented case reports. Most cases result in right sided effusions similar to hepatic hydrothorax, as seen in our case, but bilateral and left sided effusions have also been reported.^4^, ^5^ Given pleural fluid bilirubin level is not routinely tested following thoracentesis or intercostal catheter insertion, clinicians must remain astute and have a high clinical suspicion to diagnose it.

PSC is a chronic, idiopathic, cholestatic liver disease characterized by progressive inflammation, fibrosis, and stricturing of the biliary ducts, eventually leading to end‐stage liver disease. Apart from liver transplantation, there is no proven treatment to halt the progression of PSC. ERCP plays a crucial role in PSC for biliary decompression to manage cholangitis. Biliary leak is an extremely rare complication of ERCP, occurring in less than 1% of cases.^6^ Bilothorax as a consequence of ERCP is rarer, with only two other case reports identified.^7^, ^8^

Symptoms can range from minimal to severe respiratory distress mimicking acute respiratory distress syndrome. If untreated, pleuritis can develop with progression of the exudative effusion into an empyema.^9^ Given the rarity of the condition, there is no set diagnostic criteria for bilothorax. Previous studies state a pleural to serum ratio of bilirubin >1 is diagnostic of bilothorax.^5^ This was seen in our case with a pleural fluid bilirubin of 170 μmol/L, with a serum bilirubin of 140 μmol/L at the time of the initial biliary leak. However there have been case reports of bilothorax with raised pleural bilirubin, with serum ratios of <1.^9^

Nearly all reports of bilothorax in the literature identify a clear aetiology or visualize biliary leak into the pleural space. Previous reports have utilized the use of hepatobiliary iminodiacetic acid (HIDA) scan for diagnosis of biliary leak,^4^, ^7^ however due to its decreased sensitivity in patients with hyperbilirubinemia this was not performed in our case.^10^ One other case report described an inability to identify the biliary leak to account for the bilothorax, but the aetiology was likely due to anatomic variation within the common bile duct.^5^ We suspect our patient had a transient biliary tree injury during the ERCP performed for stent exchange. Due to the increased biliary pressure from her PSC, this led to biliary leak into the peritoneal cavity. This fluid subsequently migrated into the pleural space through diaphragmatic defects, aligning with the hypothesis discussed in a previous case report of bilothorax occurring following ERCP.^8^

In conclusion, our case highlights a case of bilothorax that likely occurred in the post‐operative setting following ERCP. However, the effusion accumulated was small to moderate in size and remained stable over a period of 2 months, indicating a low volume, self‐limited leak, from which the patient was asymptomatic. The effusion was aspirated as a diagnostic procedure to exclude both infection and malignancy prior to workup for liver‐transplantation. An MRCP and repeat ERCP following the diagnosis of bilothorax did not reveal a biliopleural fistula or active bile leak. Our findings highlight the importance not missing the diagnosis of bilothorax by performing thoracentesis for new effusions post ERCP and considering bilothorax in the differential diagnosis by confirmatory testing with pleural fluid bilirubin.

## AUTHOR CONTRIBUTIONS

**Hamza Azam** and **Bapti Roy**: Conception of the manuscript; literature search; drafting of the manuscript. **Mohammed Affan Guliyara**: Critical review of the manuscript, literature search. All the authors approved the final version of the manuscript.

## CONFLICT OF INTEREST STATEMENT

None declared.

## ETHICS STATEMENT

The authors declare that appropriate written informed consent was obtained for the publication of this manuscript and accompanying images.

## Data Availability

The data that support the findings of this study are available from the corresponding author upon reasonable request.
